# Indole-3-carbinol regulates microglia homeostasis and protects the retina from degeneration

**DOI:** 10.1186/s12974-020-01999-8

**Published:** 2020-11-03

**Authors:** Amir Saeed Khan, Thomas Langmann

**Affiliations:** 1 Laboratory for Experimental Immunology of the Eye, Department of Ophthalmology, Faculty of Medicine and University Hospital Cologne, University of Cologne, Joseph-Stelzmann-Str. 9, D-50931 Cologne, Germany; 2 Center for Molecular Medicine Cologne, Cologne, Germany

**Keywords:** Aryl hydrocarbon receptor, Indole-3-carbinol, Microglia, Retinal degeneration, Light damage

## Abstract

**Background:**

Retinal degenerative diseases significantly contribute to visual impairment and blindness. Microglia reactivity is a hallmark of neurodegenerative diseases including retinal cell death and immunomodulation emerges as a therapeutic option. Indole-3-carbinol (I3C) is a natural ligand of aryl hydrocarbon receptor (AhR), with potent immunomodulatory properties. Here, we hypothesized that I3C may inhibit microglia reactivity and exert neuroprotective effects in the light-damaged murine retina mimicking important immunological aspects of retinal degeneration.

**Methods:**

BV-2 microglia were treated in vitro with I3C followed by lipopolysaccharide (LPS) stimulation to analyze pro-inflammatory and anti-oxidant responses by quantitative real-time PCR (qRT-PCR) and Western blots. Nitric oxide (NO) secretion, caspase 3/7 levels, phagocytosis rates, migration, and morphology were analyzed in control and AhR knockdown cells. I3C or vehicle was systemically applied to light-treated BALB/cJ mice as an experimental model of retinal degeneration. Pro-inflammatory and anti-oxidant responses in the retina were examined by qRT-PCR, ELISA, and Western blots. Immunohistochemical staining of retinal flat mounts and cryosections were performed. The retinal thickness and structure were evaluated by in vivo imaging using spectral domain-optical coherence tomography (SD-OCT).

**Results:**

The in vitro data showed that I3C potently diminished LPS-induced pro-inflammatory gene expression of *I-NOS*, *IL-1ß*, *NLRP3*, *IL-6*, and *CCL2* and induced anti-oxidants gene levels of *NQO1*, *HMOX1*, and *CAT1* in BV-2 cells. I3C also reduced LPS-induced NO secretion, phagocytosis, and migration as important functional microglia parameters. siRNA-mediated knockdown of AhR partially prevented the previously observed gene regulatory events. The in vivo experiments revealed that I3C treatment diminished light-damage induced *I-NOS*, *IL-1ß*, *NLRP3*, *IL-6*, and *CCL2* transcripts and also reduced CCL2, I-NOS, IL-1ß, p-NFkBp65 protein levels in mice. Moreover, I3C increased anti-oxidant NQO1 and HMOX1 protein levels in light-exposed retinas. Finally, I3C therapy prevented the accumulation of amoeboid microglia in the subretinal space and protected from retinal degeneration.

**Conclusions:**

The AhR ligand I3C potently counter-acts microgliosis and light-induced retinal damage, highlighting a potential treatment concept for retinal degeneration.

## Background

Phagocytes of the retina, microglia and macrophages, have been recognized as important factors in retinal degenerative diseases. In the healthy retina, ramified microglia localize in the inner and outer plexiform layers (IPL, OPL) and scan their microenvironment with their surface receptors for cytokines, chemokines, and complement factor [1, 2]. In the diseased retina, these cells become amoeboid and migrate to subretinal (SR) space with upregulation of different pro-inflammatory molecules [2, 3]. Initially, microglia enhance their phagocytosis capacity to resolve tissue damage. However, persistent disease leads to chronically activate microglia, which may ingest not only cellular debris but also healthy photoreceptors [4]. Therefore, microglia-targeted pharmacotherapy to mitigate chronic neuro-inflammation may be a promising treatment approach in retinal degenerative diseases.

Aryl hydrocarbon receptor (AhR), a ligand-dependent transcriptional factor, is activated by various ligands [5]. Initially, AhR was identified as key regulator of xenobiotic metabolism and detoxification [6]. Inactive AhR localizes in the cytoplasm and upon binding of a ligand, the protein dissociates from chaperones and translocates into the nucleus where it binds to nuclear response element and changes gene transcription required for cellular homeostasis [6]. AhR deficiency accelerates aging in mice and triggers inflammation [7–9], and AhR ligands play an important role in immune modulation [9, 10].

In the ocular compartment, AhR knockout triggers apoptosis and inflammation in experimental autoimmune uveitis [11]. AhR deficiency also disturbs extracellular matrix biology, leading to an AMD-like pathology in mice [12]. Moreover, AhR^−/−^ mice showed a reduction in visual function, displayed an accumulation of microglia in the SR space, and had retinal pigment epithelium (RPE) abnormalities [13]. AhR^−/−^ mice also showed larger lesions in laser-induced choroidal neovascularization that mimics the wet form of AMD [14]. Furthermore, 2,2′-aminophenyl indole (2AI), a synthetic ligand of AhR could protect the RPE and retina from environmental stress [15].

Here, we hypothesized that AhR agonists may regulate microglia homeostasis and dampen experimental neurodegeneration in the retina. To test this hypothesis, we used a naturally occurring AhR agonist, indole-3-carbinol (I3C), which is abundant in green vegetables. We addressed the effects of I3C in vitro using the microglial BV-2 cell line [16] and systemically applied I3C in BALB/cJ mice exposed to an acute white-light-damage paradigm.

## Methods

### Reagents

I3C (I7256-5G) and *Escherichia coli* 0111: B4 lipopolysaccharide (LPS) were purchased from Sigma-Aldrich (St. Louis, MO, USA).

### Cell culture

BV-2 microglia were seeded in RPMI640 supplemented with 5% fetal calf serum (FCS), 1% penicillin/streptomycin, 2 mM l-glutamine and 195 nM β-mercaptoethanol at 37 °C in a humidified atmosphere of 5% CO2. The cells were pre-treated with 50 μM I3C for 4 h and then stimulated with 50 ng/ml LPS for 4 h. After treatments, cells were harvested for RNA and protein extraction. Cell supernatants were also collected for further analysis. The 661W photoreceptor-like cells were cultured in Dulbecco’s modified Eagle’s medium (DMEM) supplemented with 10% FCS, l-glutamine, and 1% penicillin/streptomycin.

### RNA isolation, reverse transcription, and quantitative RT-PCR

Total RNA was extracted from the harvested BV-2 cells using the NucleoSpin® RNA Mini Kit (Macherey-Nagel; Dueren, Germany). Total RNA was also extracted from the retinas of BALB/cJ mice using the Qiagen RNeasy Micro Kit according to the manufacturer. RNA was quantified with a NanoDrop 2000 photometer (Thermo Scientific). First-strand complementary DNA synthesis was performed using the Thermo RevertAid RT Kit (ThermoFisher Scientific; Waltham, USA). qRT-PCR analysis was performed with the Takyon™ qPCR Kit (Eurogentec Deutschland GmbH; Köln, Germany) and the Roche Probe library using the LightCycler® 480 II machine (Roche; Basel, Switzerland). Primer sequences and Roche library probe numbers were as follows: i-NOS, forward primer 5′-ctttgccacggacgagac-3′, reverse primer 5′-tcattgtactctgagggctga-3′, probe #13; IL-1ß, forward primer 5′-tcttctttgggtattgcttgg-3′, reverse primer 5′-tgtaatgaaagacggcacacc-3′, probe #38; NLRP3, forward primer 5′-ttcccagacactcatgttgc-3′, reverse primer 5′-agaagagaccacggcagaag-3′, probe #74; CCL2, forward primer 5′-catccacgtgttggctca-3′, reverse primer 5′-gatcatcttgctggtgaatgagt-3′, probe #62; IL6, forward primer 5′-gatggatgctaccaaactggat-3′, reverse primer 5′-ccaggtagctatggtactccaga-3′, probe #6; NQO1, forward primer 5′-agcgttcggtattacgatcc-3′, reverse primer 5′-agtacaatcagggctcttctcg-3′, probe #50; HMOX1, forward primer 5′-agggtcaggtgtccagagaa-3′, reverse primer 5′-cttccagggccgtgtagata-3′, probe #9; CAT1, forward primer 5′-ccttcaagttggttaatgcaga-3′, 5′-caagtttttgatgccctggt-3′, probe #34; ATPase, forward primer 5′-ggcacaatgcaggaaagg-3′, reverse primer 5′-tcagcaggcacatagatagcc-3′, probe #77. Measurements were performed in triplicates. ATP5b expression was used as a reference gene. For relative quantification, the ΔΔC method was used as implemented in the LightCycler® 480 software.

### Protein extraction, ELISA, and Western blot

RIPA buffer (150 mM sodium chloride (NaCl), 1% NP-40, 0.5% sodium deoxycholate, 0.1% sodium dodecyl sulfate (SDS), 50 mM Tris-HCl pH 7.4, supplemented with protease inhibitor cocktail (Roche)) was used to extract cell lysates. Mouse retinal tissue was homogenized in PBS using sonication. Insoluble debris was removed by centrifugation for 15 min at 16,000×*g*. Protein concentration was determined using the PierceTM Bicinchoninic Acid (BCA) Protein Assay Kit (Thermo. Scientific, Cat #23225). Twenty micrograms of cell or tissue lysates were separated by SDS-PAGE on 10% gels with PageRuler pre-stained protein ladder (Thermo Scientific; Waltham, MA, USA). Proteins were then transferred to nitrocellulose membranes (Biorad; Munich, Germany). After incubation in blocking buffer (TBS-T containing 5% nonfat dry milk) for 1 h, membranes were incubated with primary antibodies against I-NOS (dilution 1:2000 in PBS, Cat #610600, BD Transduction Laboratories™), IL-1ß (dilution 1: 200 in PBS, Cat #B122, Santa Cruz Biotechnology), p-NFκB p65 (dilution 1:500 in PBS, Cat #sc136548, Santa-Cruz Biotechnology), COX2 (dilution 1:500 in PBS, Cat #Ab1519, Abcam), NQO1 (dilution 1:500 in PBS, Cat #sc32793, Santa Cruz Biotechnology), HMOX1 (dilution 1:1000 in PBS, Cat #Ab137749, Abcam), and ß-ACTIN (dilution 1:200 in PBS, Cat #sc47778, Santa-Cruz Biotechnology). After washing steps, blots were incubated with secondary antibodies (dilution 1:4000 in PBS, Cat #P0448, Dako polyclonal goat anti-rabbit, immunoglobulins/HRP, and dilution 1:4000 in PBS, Cat #P0447, Dako polyclonal goat anti-mouse, immunoglobulins/HRP). Enhanced chemiluminescence signals were then visualized and imaged with the MultiImage II system (Alpha Innotech; Santa Clara, CA, USA). Densitometry of bands was measured using the Image J software (NIH). The concentration of CCL2 in total retinal lysates was measured by ELISA according to the manufacturer’s instructions (Mouse CCL2/JE/MCP-1 DuoSet ELISA, Cat #DY479-05, R&D Systems).

### Nitrite measurement

Nitric oxide concentrations were determined using the Griess reagent system (Promega). Briefly, 50 μl cell culture was incubated with 100 μl Griess reagent in 96-well plates. After incubation for 30 min at room temperature, absorbance was measured at 540 nm on an Infinite F200 Pro plate reader (Tecan). Nitrite concentrations were calculated as described before [17].

### Caspase 3/7 assay

To determine microglia neurotoxicity, a culture system of 661W photoreceptors cells with the microglia-conditioned medium was established. The 661W photoreceptor cells were incubated for 48 h either in their own medium or with culture supernatants from treated BV-2 cells. The 661W cells morphology was assessed by phase-contrast microscopy and cell death was determined with the Caspase-Glo® 3/7 Assay (Promega GmbH; Mannheim, Germany) as previously described [17].

### Phagocytosis assay

BV-2 cells were pre-treated with DMSO as vehicle, 50 μM I3C, vehicle +50 ng/ml LPS, and 50 ng/ml LPS + 50 μM I3C for 4 h. After treatments, 2 μl latex bead solution (polystyrene microparticles, Sigma Aldrich; St. Louis, MO, USA) was added to the wells for 4 h to determine the influence of I3C on phagocytosis. Five micrographs per well were taken using an AxioVert.A1 inverted microscope (Carl Zeiss; Germany). The phagocytic activity was determined by calculating the number of cells, which phagocytosed 10 or more latex beads.

### Scratch wound healing assay

BV-2 cells were seeded in six-well plates as 80% confluent monolayers and were wounded with a sterile 200 μl pipette tip. Thereafter, the cells were treated with vehicle, 50 μM I3C, vehicle +50 ng/ml LPS, and 50 ng/ml LPS + 50 μM I3C. Migration into the open scar was documented with microphotographs taken after 8 h of wounding using an AxioVert.A1 inverted microscope (Carl Zeiss; Germany).

### Phalloidin staining

BV-2 cells were seeded on coverslips in six-well plates. Cells were treated with vehicle, 50 μM I3C, vehicle +50 μM I3C, and 50 μM I3C + 50 ng/ml LPS. Thereafter, the cells were fixed, permeabilized with 0.1% Triton X-100 and F-actin was labeled using 0.1 μg/ml Phalloidin-TRITC (Sigma). The nuclei were stained using 4′,6-diamidino-2-phenylindole (DAPI), and coverslips were mounted onto slides using Dako fluorescent mounting medium (Dako Deutschland GmbH; Hamburg, Germany). Photos were taken with a Zeiss Imager M.2 equipped with Apotome.2 (Carl Zeiss; Germany).

### siRNA-mediated AhR gene silencing

BV-2 cells were transfected with AhR siRNA (FlexiTube siRNA containing 4 preselected siRNAs for the AhR gene, Cat #1027416, Qiagen; Hilden Germany) and the siRNA non-targeting negative control (Cat #1022076, Qiagen; Hilden Germany). All transfections were performed using lipofectamine 3000 (Invitrogen) for 6 h followed by medium refreshment and cells were further incubated for 48 h. During this incubation time, cells were treated with 50 μM I3C or 50 ng/ml LPS for 4 h.

### Animal experiments

All in vivo experiments were performed with 8-10-week-old BALB/cJ mice of both sexes. The animals were kept in an air-conditioned environment with 12-h light and dark cycle and had full access to water and food ad libitum. All experimental procedures complied with the ARVO Statement for the Use of Animals in Ophthalmic and Vision Research. The animal protocols were approved by the government office of animal welfare in North Rhine-Westphalia (Germany) (reference number 81-02.04.2019.A092). The mice received intraperitoneal injections of I3C at a dose of 15 mg/kg body weight, dissolved in DMSO or DMSO alone as vehicle control, starting 1 day before the light exposure and then once daily for the remaining days. BALB/cJ mice were dark-adapted for 16 h before light exposure. Pupil dilation was performed with 2.5% phenylephrine and 0.5% tropicamide under dim red light before the mice were exposed to bright white light (15,000 lux) for 1 h. After light exposure, the animals were kept in dark-reared conditions overnight and then maintained under normal light conditions (12-h light and dark cycle) for the remaining experimental period.

### Immunohistochemistry

Eyes were enucleated and fixed with 4% paraformaldehyde (ROTI®Histofix, Carl-Roth; Germany) for 2 h at room temperature. Retinal flat mounts were prepared and permeabilized overnight (5% Triton X-100, 5% Tween-20 in PBS). The flat mounts were incubated with BLOTTO (1% milk powder, 0.01% Triton X-100 in PBS) for 1 h to block nonspecific antigen binding. Subsequently, retinal flat mounts were incubated with primary antibody (rabbit anti-Iba1, dilution 1:1000, FUJIFILM Wako Chemicals; Neuss, Germany) overnight at 4 °C. After washing steps, the retinal flat mounts were incubated with secondary antibody (goat anti-rabbit AlexaFlour 488 (green) A11008, Life Technologies) for 1 h at room temperature. After washing steps, retinal flat mounts were mounted on a microscopic slide and embedded with fluorescence mounting medium (VECTASHIELD® HardSet™ Antifade Mounting Medium H-1400, Vector Laboratories; Burlingame, USA). Images were taken with a Zeiss Imager M.2 equipped with Apotome.2 (Carl Zeiss; Germany).

For immunohistochemical analyses of cryosections, the eyes were enucleated and fixed with 4% paraformaldehyde (ROTI®Histofix, Carl-Roth; Germany) for 2 h at room temperature. Whole eyes were then incubated with 30% sucrose overnight and embedded in optimal cutting temperature (OCT) compound, shock frozen on dry ice and then stored at −20 °C. Ten-micrometer sections were cut with a cryostat (Leica CM 3050 S, Leica biosystem; Wetzlar, Germany). Sections were rehydrated with phosphate-buffered saline (PBS) and blocked with BLOTTO (1% milk powder and 0.3% Triton X-100 in PBS) followed by overnight incubation with primary antibody (rabbit anti-Iba1, dilution is 1:500 in BLOTTO, FUJIFILM Wako Chemicals; Neuss, Germany) at 4 °C. After washing, sections were incubated with secondary antibody (goat anti-rabbit AlexaFlour 488 (green), dilution is 1:1000 in PBS, A11008, Life Technologies) for 1 h at room temperature. After washing steps, the sections were mounted with Flouromount and counterstained with Dapi (ThermoFisher Scientific; Waltham, USA). Images were taken with a Zeiss Imager M.2 equipped with Apotome.2 (Carl Zeiss; Germany).

### Optical coherence tomography (OCT)

For OCT, mice were anesthetized by intraperitoneal injection of ketamine hydrochloride (100 mg/kg body weight, Ketavet; Zoetis) and xylazine hydrochloride (10 mg/kg body weight, Rompun; Bayer HealthCare) diluted in 0.9% sodium chloride. The pupils of the mice were dilated using 2.5% phenylephrine and 0.5% tropicamide before OCT. Spectral-domain optical coherence tomography (SD-OCT) was performed on both eyes with a Spectralis™ HRA/OCT device (Heidelberg Engineering) to quantify the retinal thickness using the Heidelberg Eye Explorer Software with circular ring scans (circle diameter 3 and 6 mm), centered around the optic nerve head.

### Statistical analysis

Data were analyzed using GraphPad Prism version 7 (GraphPad Software Inc., San Diego, CA). All data were analyzed using analysis of variance (one-way ANOVA) and Dunnett’s multiple comparison test. *p* < 0.05 was considered as statistically significant.

## Results

### I3C reduces pro-inflammatory and enhances anti-oxidant gene expression in BV-2 cells

**Fig. 1:**
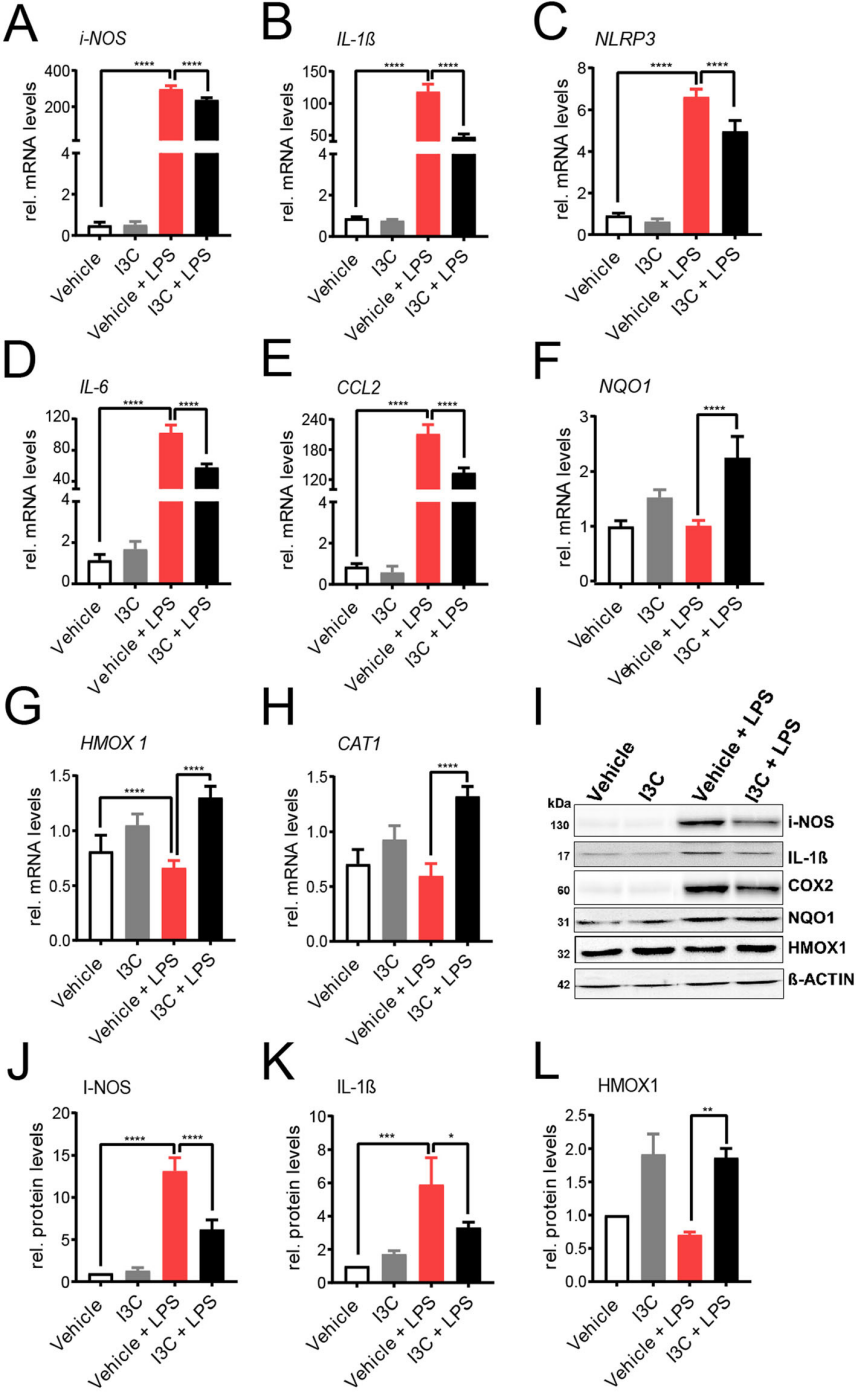
Effects of I3C on pro-inflammatory and anti-oxidant mRNA and protein levels. **a-h** BV-2 microglia cells were treated with 50 μM I3C or DMSO as a vehicle for 4 h followed by 50 ng/ml LPS for 4 h. After 8 h, mRNA expression levels of *i-NOS* **a**, *IL-1ß* **b**, *NLRP3* **c**, *IL-6* **d**, *CCL2* **e**, *NQO*1 **f**, *HMOX1* **g**, and *CAT1* **h** were analyzed by real-time PCR. **i** Western blots show i-NOS, IL-1ß, COX2, NQO1, HMOX1, and ß-ACTIN protein levels as a loading control. Mean relative protein levels are shown for i-NOS **j**, IL-1ß **k**, and HMOX1 **l**. Data show mean ± SEM out of three independent experiments for mRNA expression levels (*n* = 3/group, measured in triplicates). For Western blot, three independent experiments were performed. *p < 0.05, **p < 0.01, ***p < 0.001, ****p < 0.0001

We first examined whether treatment with the AhR ligand I3C has an effect on a selected set of pro-inflammatory markers in microglia-like BV-2 cells. The cells were pre-treated with 50 μM I3C for 4 h and then further stimulated with 50 ng/ml LPS for additional 4 h. LPS stimulation-induced mRNA levels for *inducible NO-synthase* (*i-NOS)* (Fig. 1a), *interleukin-1 beta* (*IL-1ß)* (Fig. 1b), *NOD-*, *LRR-*, *and pyrin domain-containing protein 3* (*NLRP3*) (Fig. 1c), *interleukin-6* (*IL-6*) (Fig. 1d), and *C–C motif chemokine ligand 2* (*CCL2*) (Fig. 1e) and co-treatment with I3C significantly dampened expression of these markers. Since AhR ligands are known to exert anti-oxidant effects, we also measured mRNAs for *NADPH dehydrogenase quinone 1* (*NQO1*) (Fig. 1f), *heme oxygenase 1 HMOX1* (Fig. 1g), and *catalase 1* (*CAT1*) (Fig. 1h). For each of these three genes, I3C enhanced mRNA expression levels (Fig. 1f-h). We then aimed to confirm these data using Western blots. These experiments revealed that the LPS-induced expression of i-NOS, IL 1ß, and cyclooxygenase 2 (COX2) was also reduced by I3C on the protein level, whereas HMOX1 was increased and NQO1 was unchanged (Fig. 1i-l). Taken together, the treatments with I3C reduced LPS-mediated pro-inflammatory markers and enhances antioxidant gene expression in BV-2 cells.

### I3C reduces LPS-induced nitric oxide secretion, caspase 3/7activity, and phagocytosis

**Fig. 2:**
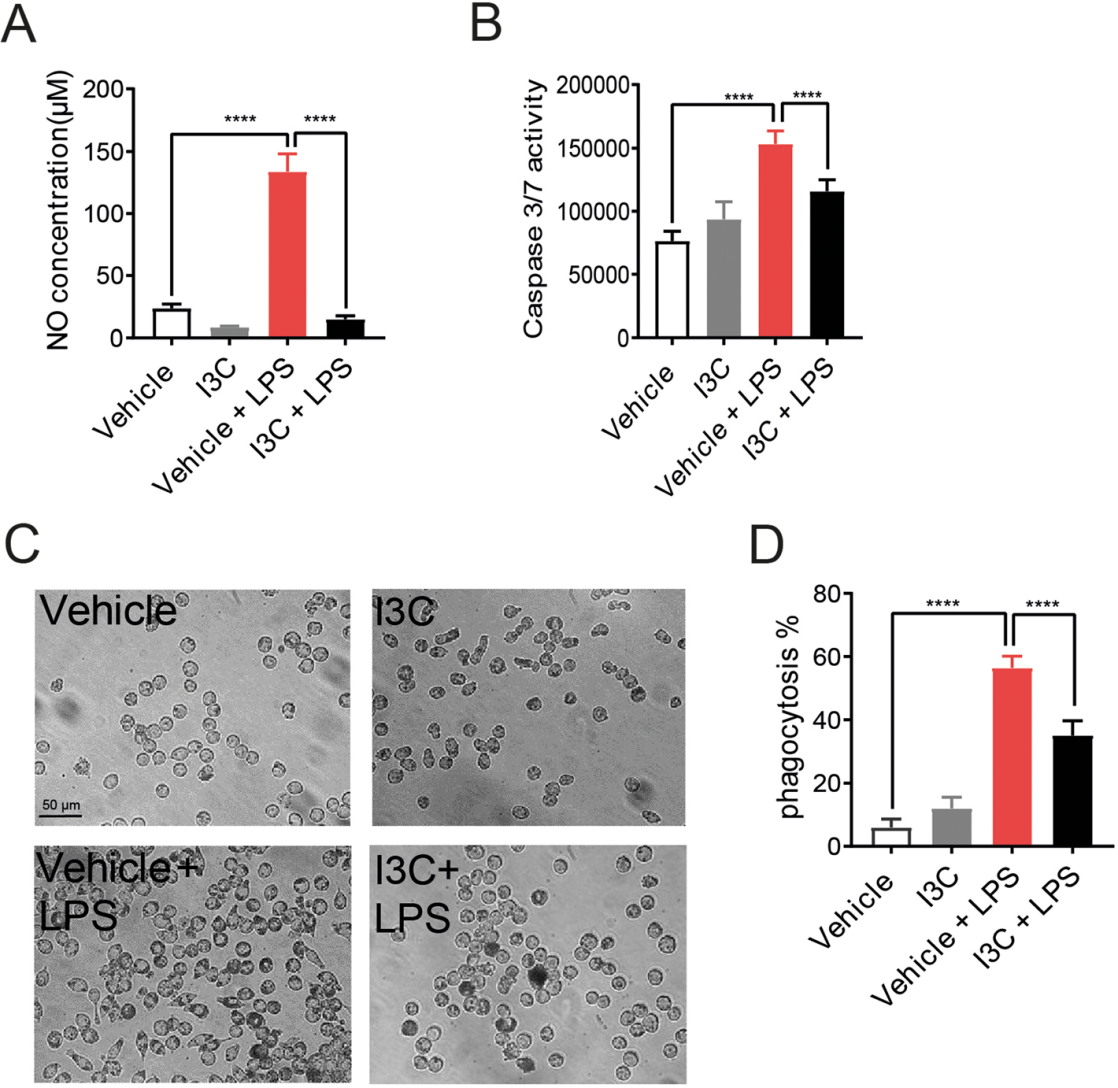
Effects of I3C on LPS-induced nitric oxide production, caspase 3/7 levels, and phagocytosis. BV-2 microglia were treated with 50 μM I3C or DMSO as a vehicle for 4 h followed by 50 ng/ml LPS. **a** Twenty-four hours later, cell culture supernatants were taken for measuring the concentration of nitric oxide by Griess assay. **b** To determine the neurotoxicity, cell culture supernatant from BV-2 microglia were transferred to 661W photoreceptor cells. After 48 h of incubation of 661W cells with conditioned medium from BV-2 cells, caspase 3/7 activity was measured to determine apoptotic cell death. **c** Representative images of microglia incubated with latex beads for 4 h after treatments. **d** Relative phagocytosis of BV-2 cells was calculated by counting the cells having more than 10 latex beads. Data show mean ± SEM out of three independent experiments (*n* = 3/group, measured in triplicates) with ****p < 0.0001

We next analyzed three functional parameters of microglia activity, namely, NO secretion, neurotoxicity, and phagocytosis. In accordance with our mRNA and protein data on i-NOS expression, I3C also significantly reduced the LPS-triggered NO secretion from BV-2 cells (Fig. 2a).

To test the influence of I3C on microglia neurotoxicity, the cone-derived 661W photoreceptor cell line was used and the cells were incubated for 48 h with conditioned media from BV-2 microglia and caspase-related apoptotic cell death was analyzed. The 661W cells cultured in LPS-activated microglia supernatants showed significantly higher caspase 3/7 activity as compared to the vehicle (Fig. 2b). This increase in caspase 3/7 activity was significantly diminished by I3C treatment (Fig. 2b).

To study the effects of I3C on phagocytosis, BV-2 cells were treated with I3C and LPS for 4 h and then latex polystyrene beads were added for another 4 h. LPS strongly induced the number of phagocytosed beads compared to vehicle (Fig. 2c-d), whereas I3C treatment significantly reduced the number of phagocytosed beads (Fig. 2c-d).

### I3C reduces LPS-induced migration and induces filopodia formation in BV-2 cells

**Fig. 3:**
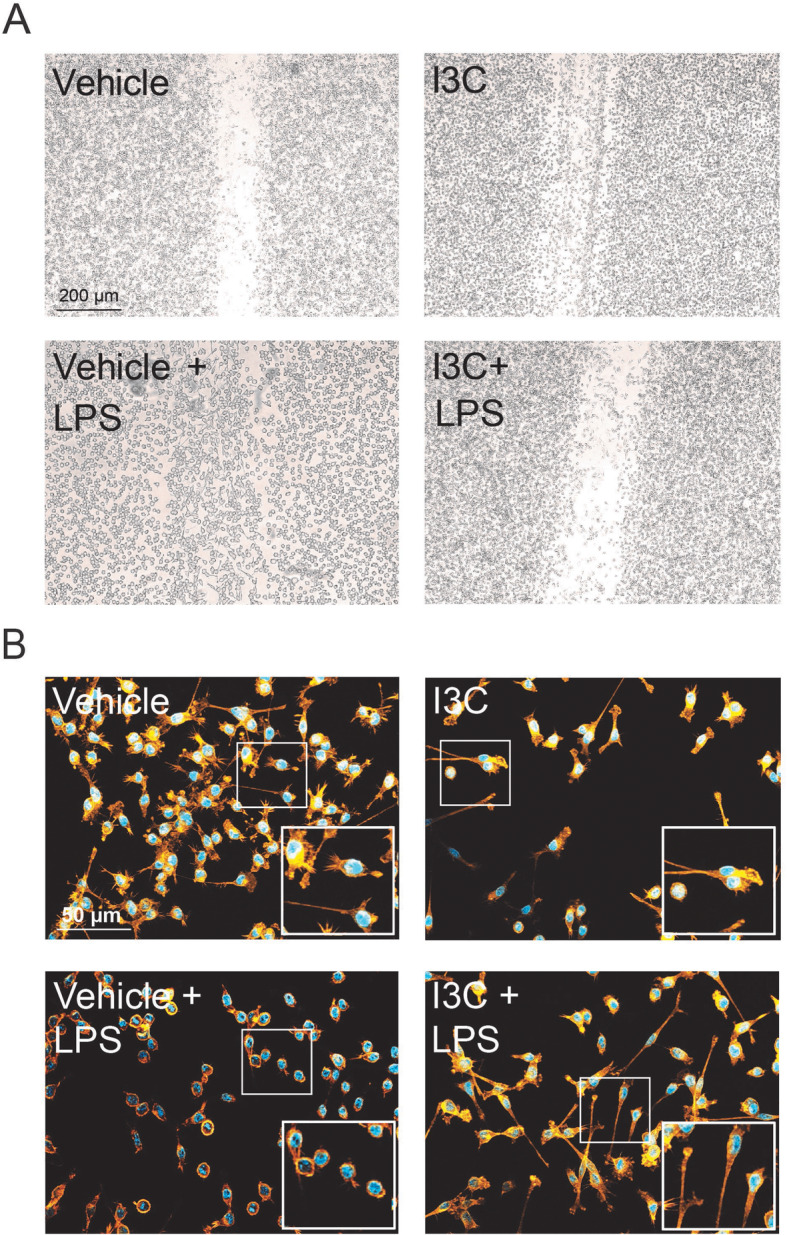
Effects of I3C on LPS-induced migration and morphology of BV-2 microglia. BV-2 microglia cells were treated with 50 μM I3C or DMSO as a vehicle for 4 h followed by 50 ng/ml LPS after scratch with a pipette tip. **a** Representative images of microglia migration toward the scratch area after 8 h. **b** Representative images of the BV-2 cell morphology stained with phalloidin and DAPI. (*n* = 3/group, measured in triplicates)

To study the influence of I3C on microglia migration, wound-healing scratch assays were conducted in BV-2 cells. LPS induced the migration of BV-2 microglia cells into the scratch (Fig. 3a), and I3C effectively blocked this migration response (Fig. 3a). The morphology of microglia is another hallmark of activation and hence we stained the F-actin cytoskeleton with phalloidin-TRITC in the different culture conditions. LPS clearly triggered a change in morphology from ramified to an amoeboid shape (Fig. 3b). Incubations in the presence of I3C clearly transformed the BV-2 cells to a highly ramified phenotype with most cells containing long filopodia (Fig. 3b). Taken together, I3C reduces LPS-induced migration and induces filopodia formation in BV-2 microglia cells.

### The AhR pathway is involved in the in vitro effects of I3C on pro-inflammatory gene expression

**Fig. 4:**
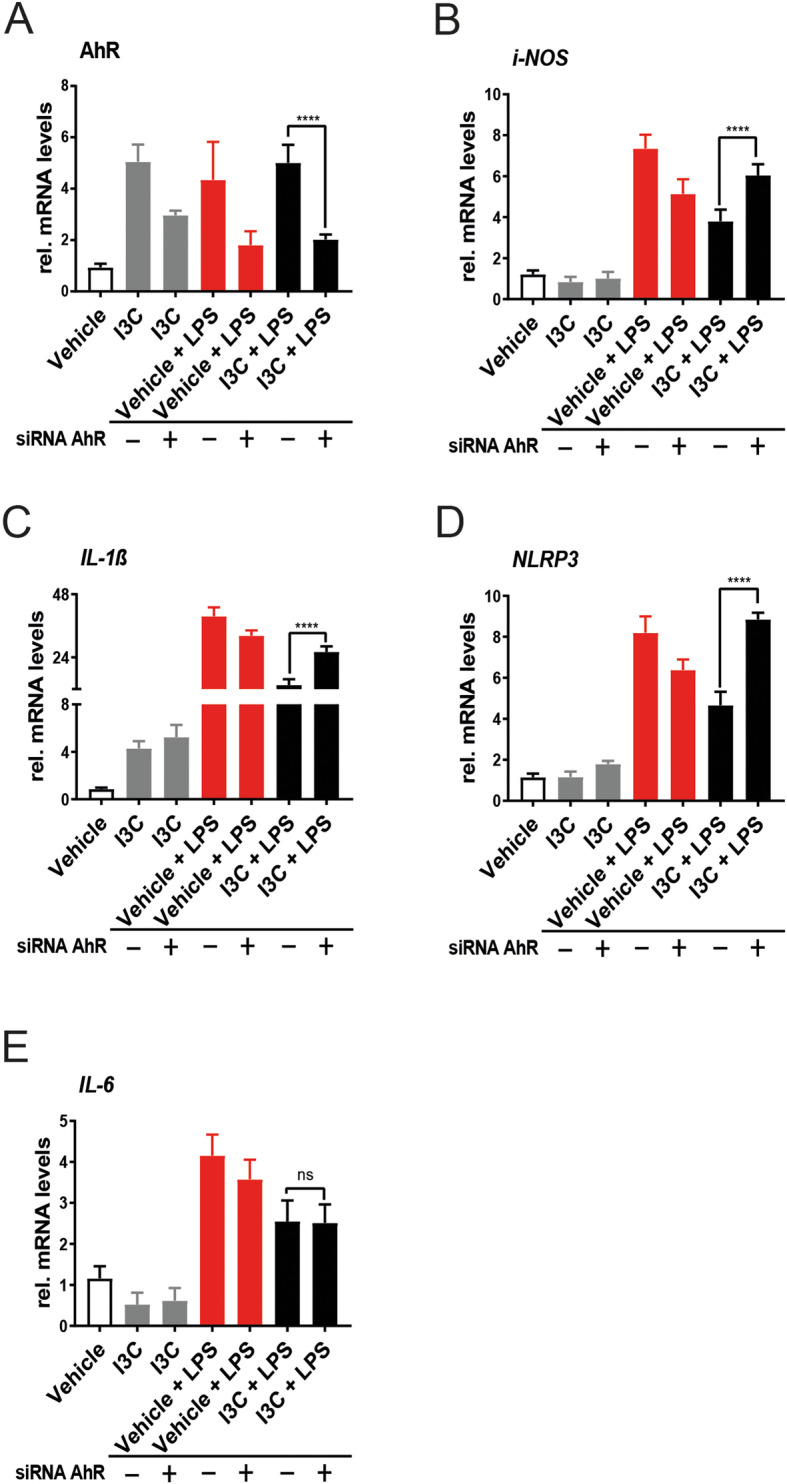
Effects of AhR knockdown on pro-inflammatory markers in BV-2 microglia. Cells were transfected with AhR-siRNA for 6 h followed by treatment with I3C and LPS. **a-e** mRNA expression levels of *AhR* **a**, *i-NOS* **b**, *IL-1ß* **c**, *NLRP3* **d**, and *IL-6* **e** were analyzed by real-time PCR. Data show mean ± SEM out of three independent experiments (*n* = 3/group, measured in triplicates) with ****p < 0.0001

To further investigate the AhR pathway in modulation of pro-inflammatory gene expression, siRNA-mediated knockdown of AhR was performed in BV-2 microglia. The cells were transfected with AhR siRNA or non-targeting negative control siRNA. We detected that the mRNA level of AhR itself was significantly downregulated by AhR siRNA (Fig. 4a). Treatment of AhR siRNA-transfected cells with I3C plus LPS caused a relative increase in *i-NOS* (Fig. 4b), *IL-1ß* (Fig. 4c), and *NLRP3* (Fig. 4d) transcript levels compared to the siRNA negative control. In contrast, *IL-6* mRNA expression levels were not changed in the presence of AhR siRNA (Fig. 4e). Thus, AhR knockdown partially prevented the previously detected anti-inflammatory effects of I3C on LPS-activated BV-2 cells.

### I3C treatment regulates pro-inflammatory and antioxidant genes in the light-damage model of murine retinal degeneration

**Fig. 5:**
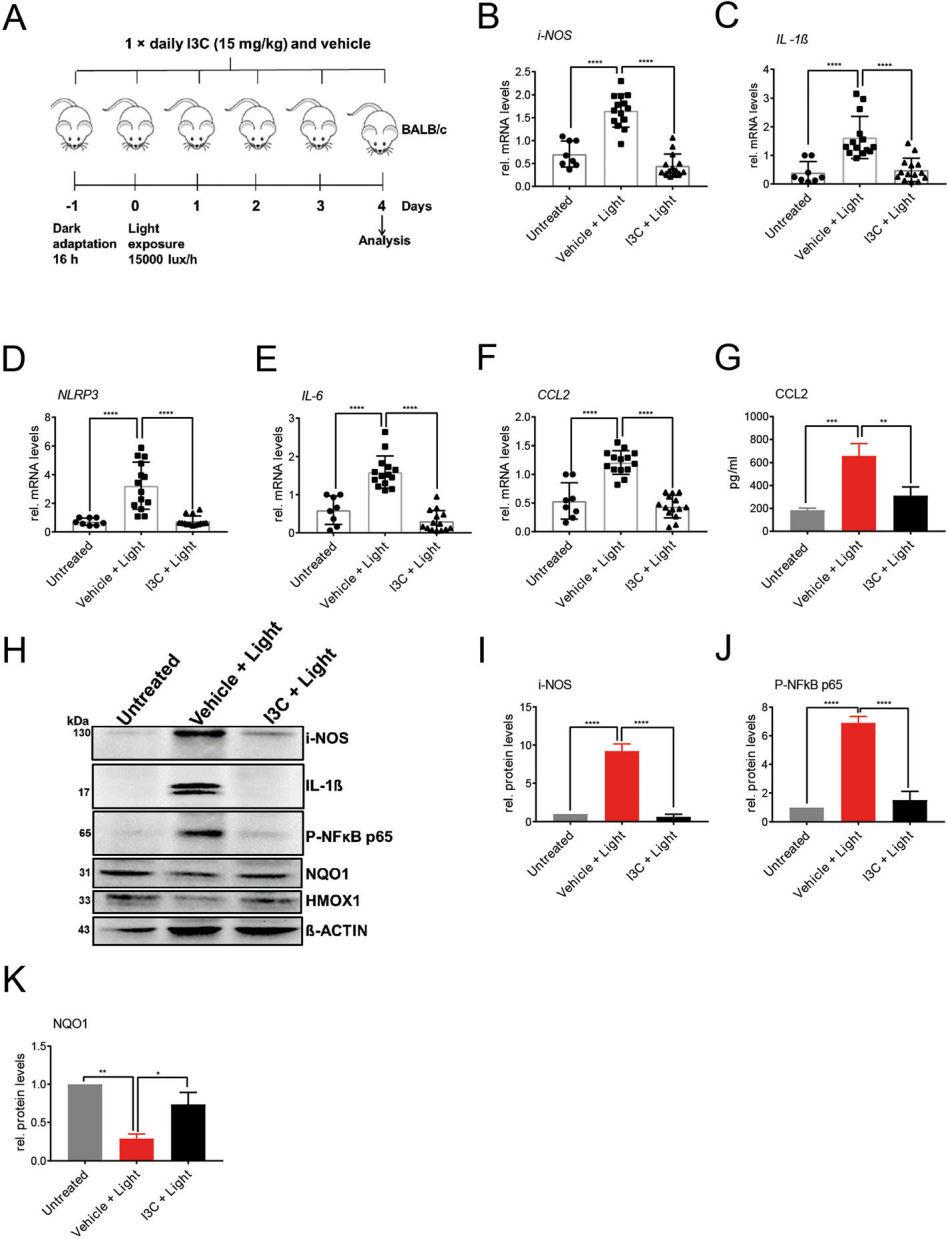
Effects of I3C on mRNA and protein levels in light-challenged BALB/cJ mice. **a** Schematic overview of experimental design. Eight to ten-week-old BALB/cJ mice of both sexes were used. Mice were dark-adapted for 16 h before the mice were exposed to white light with an intensity 15,000 lux for 1 h. The animals received intraperitoneal injections of 15 mg/kg I3C or vehicle (DMSO), 1 day before the light exposure and once daily for the remaining 3 days. Four days after the light exposure, **b-f** mRNA expression levels of *i-NOS* **b**, *IL-1ß* **c**, *NLRP3* **d**, *IL-6* **e**, and *CCL2* **f**, were analyzed by real-time PCR. Four days after the light exposure, retinal lysates were extracted to perform ELISA of CCL2 **g** and Western blots **h** detecting i-NOS, IL- 1ß, p-NFKB65, NQO1, and HMOX1 protein levels. Beta-actin served as loading control. Relative protein levels for i-NOS **i**, p-NFkBp65 **j**, and NQO1 **k** were determined by densitometry. Data show mean ± SEM out of three independent experiments for mRNA expression levels (untreated *n* = 8 retinas, vehicle + light *n* = 14 retinas, light + I3C treatment *n* = 14 retinas, where each dot represents one retina). For Western blots and ELISA three independent experiments were performed (one retina per group) with *p < 0.05, **p < 0.01, ***p < 0.001, and ****p < 0.0001

We next aimed at analyzing the effects of I3C in the murine retina subjected to an established light damage paradigm of retinal degeneration. Light-sensitive BALB/cJ mice were dark-adapted for 16 h before the mice were exposed to white light with an intensity of 15,000 lux for 1 h. The animals received intraperitoneal injections of 15 mg/kg I3C or DMSO as a vehicle, the day before the light exposure, and then once daily for the remaining 3 days (Fig. 5a).

Four days after light exposure, retinal mRNA expression levels of the pro-inflammatory markers *i-NOS* (Fig. 5b), *IL-1ß* (Fig. 5c), *NLRP3* (Fig. 5d), *IL-6* (Fig. 5e), and *CCL2* (Fig. 5f). Whereas relative expression of these genes changed in light exposed and vehicle-treated retinas, I3C treatment significantly reversed these effects (Fig. 5b-f).

We next verified these mRNA expression differences on the protein level and further included two antioxidant proteins, NQO1 and HMOX1, that are mainly regulated on protein level. The effects on retinal CCL2 protein expression were verified using ELISA, which showed that I3C reduced light-induced CCL2 secretion (Fig. 5g). Western blots further revealed that I3C also reduced light-damage induced i-NOS, IL-1ß, and p-NFKBp65 protein levels in the retina (Fig. 5h-j), whereas protein levels for the antioxidant genes NQO1 and HMOX1 were upregulated in I3C-treated animals (Fig. 5h, k).

### I3C prevents microglia reactivity in the light-damaged retina

**Fig. 6:**
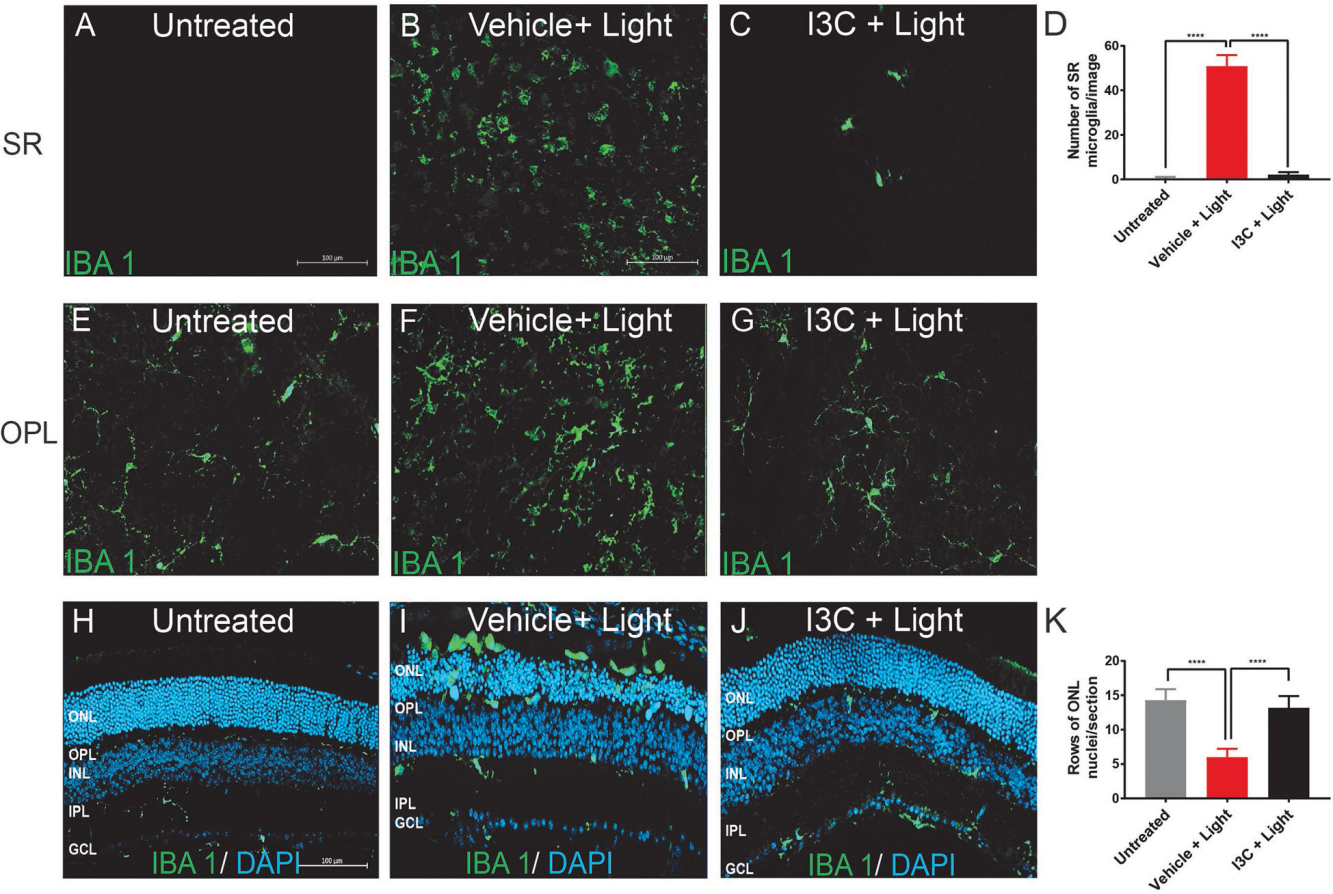
Effects of I3C in retinal microglia reactivity elicited by light in BALB/cJ mice. **a-g** Representative photomicrographs of IBA 1 stained microglia (green) of retinal flat mounts. Microglia in the subretinal space (SR) and outer plexiform layer (OPL) of control **a**, **e**, vehicle + light **b**, **f**, and I3C + light **c**, **g** treated mice. **d** Total IBA 1+ cells were counted in the subretinal space (untreated *n* = 20 retinas, vehicle + light = 20 retinas, and I3C + light *n* = 20 retinas). **h-j** Representative photomicrographs show retinal sections of control **h**, vehicle + light **i**, and I3C + light **j** treated mice stained with IBA 1 (green) and DAPI (blue). **k** For quantification of ONL thickness in cryosections, rows of photoreceptor nuclei were counted (untreated *n* = 10 retinas, vehicle + light *n* = 10 retinas, I3C + light *n* = 10 retinas). Data show mean ± SEM with ****p < 0.0001. ONL outer nuclear layer, OPL outer plexiform layer, INL inner nuclear layer, IPL inner plexiform layer, GCL ganglion cell layer

To assess the effects on retinal microglia in vivo, retinal flat mounts of light-challenged and I3C-treated mice were stained with the marker IBA1. We found that retinas of untreated mice show no microglia in the subretinal (SR) space (Fig. 6a), whereas vehicle + light-treated animals showed abundant amoeboid microglia in this area (Fig. 6b). Thus, accumulation of amoeboid microglia in the SR space was prevented by I3C treatment (Fig. 6c, d). The outer plexiform layer (OPL) of untreated control animals showed mainly ramified microglia (Fig. 6e). In contrast, vehicle-treated and light-exposed mice had less ramifications and showed a more amoeboid morphology (Fig. 6f). Again, these signs of microglia reactivity were prevented by I3C therapy (Fig. 6g).

We also analyzed the localization of IBA1-positive cells in retinal layers using cryosections. In untreated animals, mainly ramified microglia were detected in both plexiform layers (Fig. 6h). In contrast, amoeboid microglia were present in the ONL of light-damage and vehicle-treated mice (Fig. 6i). Notably, I3C nearly fully prevented this light-induced amoeboid microglia accumulation (Fig. 6j). Furthermore, the DAPI staining of cryosections of light-damage and vehicle-treated mice indicated a significant cell loss in the ONL, which was prevented by I3C (Fig. 6h-k).

### I3C preserves retinal thickness after light exposure

**Fig. 7:**
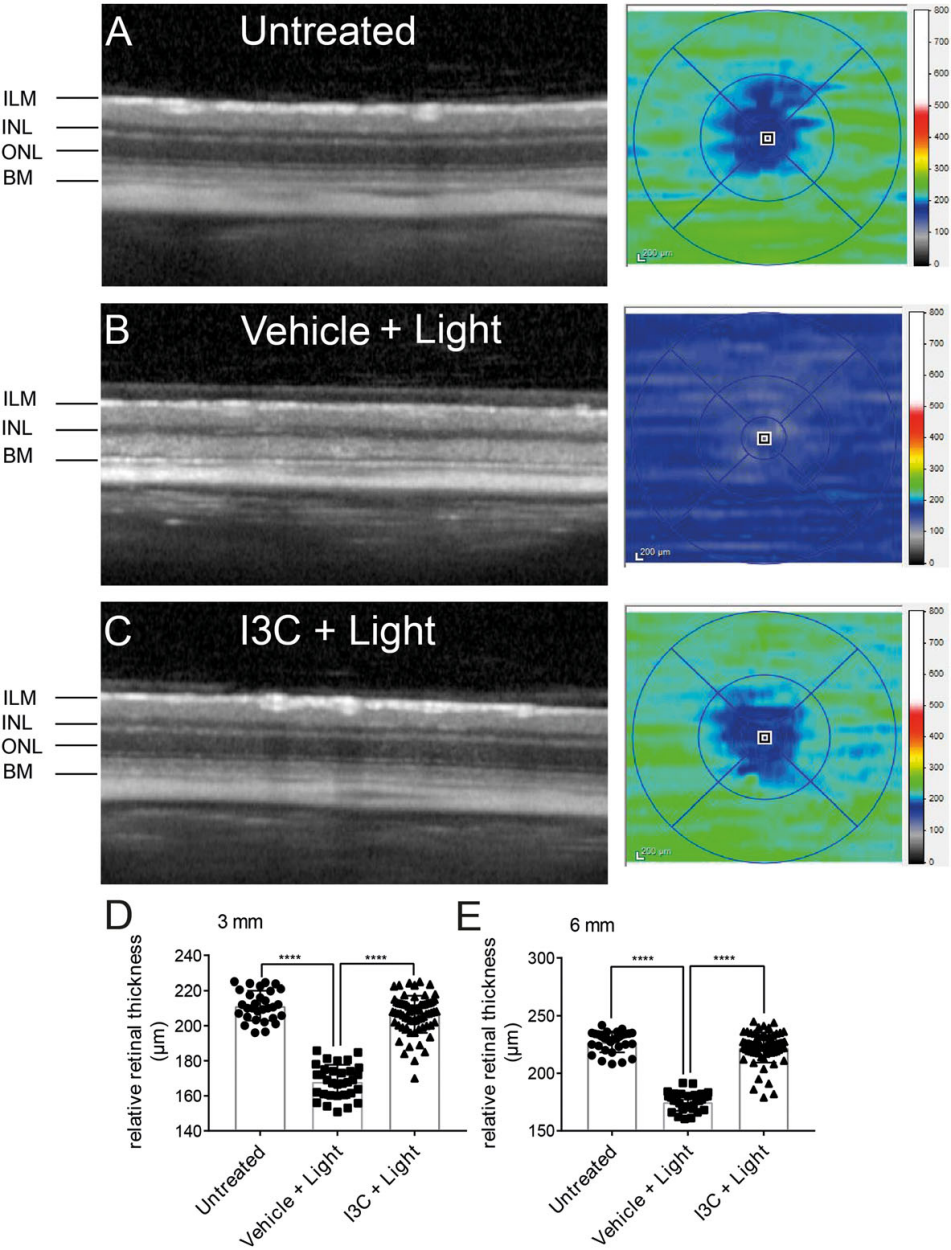
Effects of I3C on retinal thickness in light-damaged BALB/cJ mice. **a-c** Four days after light damage, SD-OCT was performed to analyze the changes in retinal thickness displaying heat maps of **a** untreated, **b** vehicle + light, and **c** I3C + light-treated mice. **d-e** Relative retinal thickness (μm) in 3 mm and 6 mm areas were calculated using the SD-OCT software, where one data point represents the average thickness of the central retina. Data show mean ± SEM (untreated *n* = 30 eyes, vehicle + light *n* = 32 eyes, light + I3C treatment *n* = 70 eyes). ****p < 0.0001

Finally, we analyzed the effects of I3C on retinal degeneration 4 days after acute light exposure. In vivo imaging with optical coherence tomography (OCT) showed strong retinal thinning and nearly complete loss of the ONL in light-exposed mice that received vehicle treatment compared to animals not subjected to light damage (Fig. 7a, b). Treatment with I3C clearly prevented this retinal degeneration (Fig. 7c), as confirmed by repeated quantitative analyses of the central retinal thickness within 3 mm and 6 mm ring scan areas (Fig. 7d, e).

## Discussion

Recent studies showed that AhR is important for the maintenance of photoreceptor and RPE homeostasis [13, 15]. To our knowledge, we here show for the first time that AhR also regulates microglia homeostasis in the degenerating retina and that its natural ligand I3C protects from acute light damage, a model system that mimics certain aspects of human retinal degeneration.

We started our study with in vitro experiments using BV-2 microglia treated with LPS and I3C. BV-2 is an established murine microglia cell line [16]. Despite its limitation as an immortalized cell type, BV-2 cells represent a good alternative for primary microglia as gene expression profiling showed that LPS-treated BV-2 cells shared 90% of genes with primary microglia [18–20]. We found here that I3C potently reduced LPS-triggered gene and protein expression of well-established pro-inflammatory transcripts [17, 21, 22]. Conversely, I3C induced anti-oxidant gene expression, which is consistent with previous findings that I3C has antioxidant activity [23]. Our data are in line with previous findings that I3C can block LPS-induced IL-1β, IL-6, and NO in RAW264.7 macrophage cells [24]. Previous findings also showed that I3C mediates apoptosis in different cancer cells [25–27]. However, our results indicate that I3C can reduce neurotoxic effects of activated microglia on 661W photoreceptor cells. Despite the limitation that 661W cells are derived from a retinal tumor and express mainly cone-specific markers [28], the cells have been documented as reliable in vitro model to study molecular pathways of neuronal apoptosis [29, 30].

In this study, LPS induced the phagocytosis of latex beads in BV-2 cells, consistent with previous findings [20, 21, 31]. We also found that I3C significantly reduced this phagocytic capacity. Phagocytosis assays with latex beads have clear limitations and may only serve as a proxy for the real situation in neuronal tissue. Clearly, in vivo phagocytosis requires multiple cellular interactions and complex molecular signaling events in microglia that are absent in cell culture phagocytosis assays [32, 33]. Nevertheless, our previous data showed a good correlation of microglial phagocytosis rates measured with latex beads and apoptotic photoreceptor debris [21]. Our study further revealed that I3C reduced LPS-induced migration and reversed the typical LPS-triggered amoeboid phenotype of BV-2 microglia [21, 34]. These data support the finding that AhR is a master regulator of cellular phagocytosis, migration, and morphology [35–37].

We also investigated the specificity of I3C using siRNA-mediated knockdown of AhR in BV-2 microglia. These data revealed that knockdown of AhR prevents the I3C-mediated decrease of i-NOS, IL-1ß, and NLRP3. However, we found that AhR knockdown did not alter IL-6 mRNA. This suggests that AhR is not directly involved in LPS-induced IL-6 gene regulation. In accordance with our results, AhR activation downregulated NLRP3 and IL-1ß mRNA levels in peritoneal macrophages and siRNA-mediated knockdown of AhR reversed this effect [38].

The in vitro data of I3C effects lead us to the analysis of microglia in a white-light-damage paradigm of retinal degeneration. Light damage is a convenient and reproducible method for inducing synchronized photoreceptor damage [39]. However, differences in the effects of neuroprotective agents between light damage and genetic mouse models have been documented [40]. Therefore, effects seen in this model cannot be transferred to retinal degenerative diseases in general. Our results clearly showed that I3C inhibited light-induced pro-inflammatory gene expression in vivo as i-NOS, IL-1ß, NLRP3, and IL-6 transcripts were significantly diminished in I3C-treated animals. These data are in agreement with other neuroinflammatory and ocular disorders. Thus, I3C prevented ischemic reperfusion-induced inflammation and improved neurobehavioral symptoms in a cerebral ischemic stroke model [41, 42]. AhR agonists also reversed pro-inflammatory gene expression in experimental autoimmune uveitis [11]. Here, we found that I3C prevented light-induced CCL2 expression in the retina. Upregulation of CCL2 in light-exposed animals has been documented by others before [43, 44]. We further showed that I3C reduced light-induced IL-1ß and p-NFkBp65 levels in the retina. Likewise, it has been shown that AhR activation limits IL-1ß and NFkB in microglia [38, 45, 46].

We also noticed that I3C upregulated NQO1 and HMOX1 protein levels in the retina of light-treated animals. Consistent with our data, 2AI, a synthetic AhR ligand enhanced the antioxidant battery of genes in the retina [15]. AhR is obviously necessary for normal immune physiology as AhR-deficient mice display a highly inflammatory phenotype with high levels of oxidative stress in the retina [47, 48].

Here, we used the AhR ligand I3C in a retinal light damage model and showed previously unknown effects on microglia reactivity. We have selected I3C as it is a known natural anti-inflammatory agent that can act on neuronal tissues. For example, it reduced clonidine-induced neurotoxicity in rats, and enhanced antioxidant levels in experimental models of Parkinson’s disease [49, 50]. I3C is rapidly absorbed and distributed in the blood, liver, kidney, lung, heart, and brain [51]. AhR is expressed by various retinal cell types including microglia, RPE, photoreceptors, and retinal ganglion cells [15, 52, 53]. Previous studies highlighted the potential role of AhR in retinal neuroprotection without focusing on microglia [12, 13, 15]. Together with these published data, our findings suggest that AhR activation in the damaged retina may have a dual beneficial effect on RPE as well as microglia homeostasis.

## Conclusions

Our results highlight an important function of AhR and its ligand I3C in regulating retinal microglia homeostasis. The observed anti-inflammatory and neuroprotective effects suggest that AhR may be a potential therapeutic target for retinal degeneration.

## Data Availability

The data supporting the findings of this study are available from the corresponding author upon request.

## Abbreviations

2AI: 2,2′-aminophenyl indole
AhR: Aryl hydrocarbon receptor
AMD: Age-related macular degeneration
CAT1: Catalase 1
CCL2: C–C motif chemokine ligand 2
COX2: Cyclooxygenase 2
FCS: Fetal calf serum
HMOX1: Heme oxygenase 1
i-NOS: Inducible NO synthase
I3C: Indole-3-carbinol
IL-6: Interleukin-6
IPL: Inner plexiform layer
LPS: Lipopolysaccharide
NLRP3: NOD-, LRR-, and pyrin domain-containing protein 3
NO: Nitric oxide
NQO1: NADPH dehydrogenase quinone 1
OPL: Outer plexiform layer
qRT-PCR: Quantitative real-time PCR
SD-OCT: Spectral domain-optical coherence tomography
siRNA: Small interfering RNA
SR: Subretinal space
